# ST-segment elevation after blunt chest trauma: myocardial contusion with normal coronary arteries or myocardial infarction following coronary lesions

**DOI:** 10.11604/pamj.2017.28.26.12272

**Published:** 2017-09-13

**Authors:** Amine Ghalem, Hanane Boussir, Kamal Ahsayan, Nabila Ismaili, Noha El Ouafi

**Affiliations:** 1 Department of Cardiology, Mohammed VI University Hospital, 60049 Oujda, Morocco; 2 Department of Anesthesia and Intensive Care, Mohammed VI University Hospital, 60049 Oujda, Morocco

**Keywords:** Blunt chest trauma, myocardial contusion, myocardial infarction, coronary angiography

## Abstract

Cardiac lesions secondary to blunt chest trauma vary from insignificant arrhythmias to fatal cardiac rupture. Of these, a distinction remains difficult; face to ST-segment elevation on ECG with positive cardiac biomarkers, is it a myocardial contusion or a genuine myocardial infarction (MI) secondary to coronary lesions? We report the case of a patient admitted for multiple trauma. Initial assessment showed an ST segment elevation on ECG, along with multiple fractures and abdominal injuries. We would like to discuss, through this case, the similarities and the differences between myocardial infarction due to coronary lesions and myocardial contusion in a traumatic context, but also emphasize the difficulty of striking the right balance between thrombotic and bleeding risks in this situation, and insist on the importance of a multidisciplinary and collegial reflexion so we can offer these patients the best care there is.

## Introduction

Cardiac lesions secondary to blunt chest trauma can vary from insignificant arrhythmias to fatal cardiac rupture [1]. In this context, a distinction remains challenging when facing an ST-segment elevation on the ECG with positive cardiac biomarkers: myocardial contusion or genuine myocardial infarction (MI) secondary to coronary lesions. We report the case of a patient admitted to our hospital after a multiple trauma with ST segment elevation on ECG. We would like to discuss, through this case, the similarities and the differences between these two entities in the context of multiple trauma, but also highlight the difficulty of managing the fragile balance between thrombotic and bleeding risks in this situation and insist on the importance of a multidisciplinary approach to these patients.

## Patient and observation

**Figure 1:**
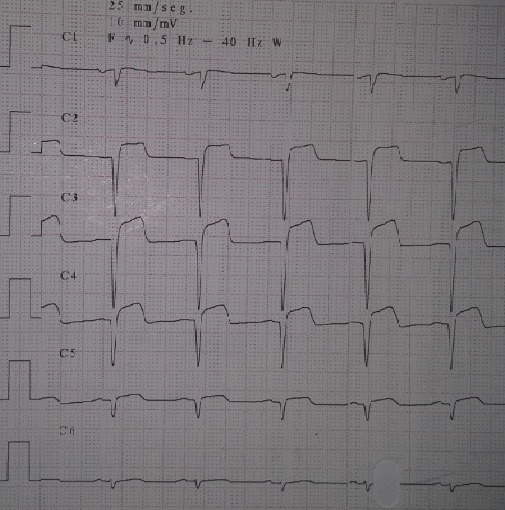
ECG (precordial leads) showing St-segment elevation and pathologic Q waves from V1 to V6

**Figure 2:**
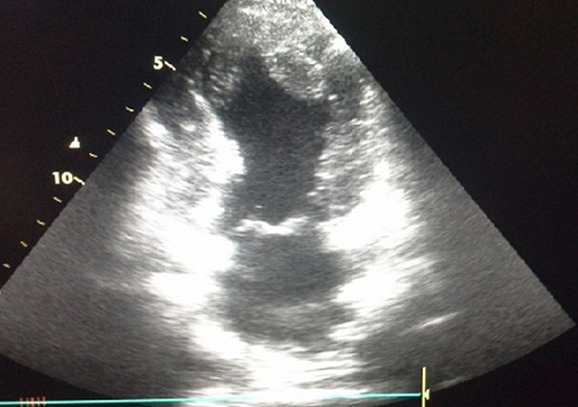
Transthoracic echocardiography: an apical two chamber view showing two enormous intraventricular apical thrombi

**Figure 3:**
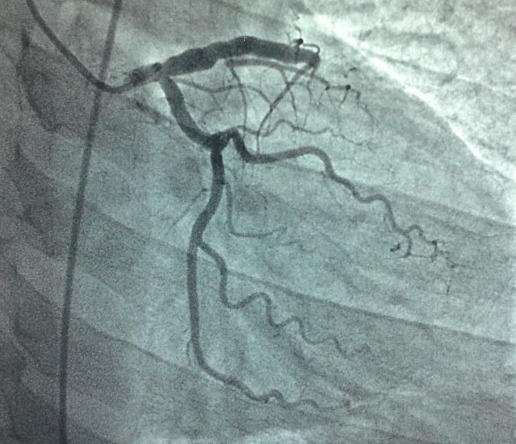
Coronary angiography (RAO 16°/ CAU 25°) showing an occlusion of the left anterior descending coronary artery

A 54 years old male, with no medical history but an active smoking, was admitted to the emergency department (ED) after a single motor vehicle crash. On admission, the patient complained of vague chest and shoulder pain. Physical examination revealed an alert, hemodynamically stable patient. Cardiac examination revealed regular heart rhythm with no murmur nor pericardial rubs. A tenderness of the right collarbone and a bilaterally diminished breath sounds were noted. Radiologic workup revealed a bilateral pleural effusion, a fracture of the right collarbone, a hepatic contusion, a right adrenal hematoma and a fracture of the left iliac wing. The ECG showed an ST-segment elevation in the precordial leads, with Q waves in V1, V2, V3 and V4 (Figure 1). Transthoracic echocardiography (TTE) revealed an anterior, anteroseptal and inferoseptal akinesis, as well as moderately decreased left ventricular ejection fraction (LVEF: 40%) and no pericardial effusion. Laboratory tests result demonstrated a rhabdomyolysis and positive troponins (Troponin Ic: 0.78 ng/mL). Given the hemorrhagic risk, antiplatelet therapy wasn't initiated. We started prophylaxis for deep venous thrombosis/pulmonary embolism (DVP/PE), proton pump inhibitor (PPI) and enteral nutrition. Fractures were treated orthopedically. As for the abdominal injuries, no specific treatment was needed. The patient was closely monitored in the cardiac intensive care unit. Three days after his admission, the patient presented an upper gastrointestinal bleeding, secondary to an ulcerative bulbitis and congestive gastritis, which we treated by intensifying the PPI, transfusion of packed red blood cells and a Helicobacter pylori eradication protocol with favorable evolution. A TTE revealed two intraventricular thrombi afterward (Figure 2). Given the important thrombotic risk, in consultation with the intensive care specialists and gastroenterologists, therapeutic anticoagulation was started. Initially, the patient was given unfractionated heparin, then, once we have been reassured concerning the absence of hemorrhagic diathesis, we switched to Low molecular weight heparin and vitamin K antagonist. Meanwhile, we performed a coronary angiography (CAG) that revealed an occlusion of the left anterior descending coronary artery within an atheromatous coronary network (Figure 3).

## Discussion

Thoracic injuries are the third most common cause of trauma. It occurs typically in road accidents affecting primarily adult males. Of these Chest trauma, between 3 and 56% of patients will have a myocardial contusion and 5% will suffer a myocardial infarction (MI) [1]. These MI are secondary to coronary arteries lesions, which can have several mechanisms: dissection, sub-intimal hemorrhage, intraluminal thrombosis, spasm, rupture, hemorrhage within a preexisting atherosclerotic plaque, or even a compression by an epicardial hematoma [2]. In our patient, the initial discussion was the following: given the ST-segment elevation, was it a myocardial contusion with otherwise normal coronary arteries? Or a MI due to a coronary lesion? And in the latter case, did the MI cause the accident or was it a consequence? History taking and physical examination have not been able to determine the exact circumstances of the crash. The ECG abnormalities noted can be seen in either myocardial contusion or MI [3]. As to biology, CPK and CPK MB tests have no interest in practice to search for myocardial injury in the context a multiple trauma due to a possible rhabdomyolysis. Troponin I or T tests are more sensitive and specific, however, positive troponins can also be observed in both situations, although the enzymatic movements are more important in MI [1, 3]. The TTE revealed left ventricular wall motion abnormalities, a common feature to both entities. It is worth noting that limitation in acquiring adequate windows for TTE in the setting of blunt chest trauma makes transesophageal echocardiography (TEE) a valuable tool in hemodynamic assessment [4]. In a patient victim of blunt chest trauma, electrical signs suggestive of MI and elevation of cardiac biomarkers (mainly troponin) should spur an emergency coronary angiography (CAG) [3]. We performed CAG, ten days after patient's admission because of injuries exposing to exsanguination, whose results supported the diagnosis of MI due to coronary lesions. The question that arose soon after patient's admission was treatment. Should we have started anticoagulation and antiplatelet therapy? To our knowledge, there is no established protocol for the management of MI in multiple trauma patients apart from the classical indication of urgent revascularization, which can be done in this context of trauma only by angioplasty, fibrinolysis being contraindicated [3, 5, 6]. Therefore, the decision must be made on a case by case basis taking into consideration the fragile balance between bleeding and thrombotic risks. In our case, after multidisciplinary discussion, we decided to refrain from antithrombotics initially, to give the patient DVP/PE prophylaxis, but the discovery of left ventricular thrombi has prompted us to start therapeutic anticoagulation. We also decided to withheld statins as there is conflictual evidence whether their use is safe/beneficial or harmful after trauma [7]. Lastly, one question remained unanswered, that of the timing of MI. Did the MI cause the accident? Or was it the contrary?

## Conclusion

In multiple trauma setting, myocardial lesions must be searched for systematically. ECG and repeated troponins must be obtained promptly. A normal ECG and troponins eliminate myocardial injury. If the latter is suspected, a TTE becomes necessary. Coronary angiography, in current practice, remains a relatively available imaging technique, which can help differentiate myocardial contusion with otherwise normal coronary arteries and myocardial infarction secondary to coronary injury. The complexity of decision making, in this type of cases, requires a multidisciplinary discussion to assess the benefit/risk of the therapeutics.

## Competing interests

The authors declare no conflicts of interests.
